# Regulated necrosis, a proinflammatory cell death, potentially counteracts pathogenic infections

**DOI:** 10.1038/s41419-022-05066-3

**Published:** 2022-07-22

**Authors:** Guangzhi Zhang, Jinyong Wang, Zhanran Zhao, Ting Xin, Xuezheng Fan, Qingchun Shen, Abdul Raheem, Chae Rhim Lee, Hui Jiang, Jiabo Ding

**Affiliations:** 1 Institute of Animal Sciences of Chinese Academy of Agricultural Sciences, Beijing, 100193 China; 2 Shenzhen Bay Laboratory, Institute of Infectious Diseases, Shenzhen, 518000 China; 3 Institute of Respiratory Diseases, Shenzhen People’s Hospital, The Second Clinical Medical College, Jinan University, Shenzhen, 518020 Guangdong China; 4 Department of Molecular and Cell Biology and Cancer Research Laboratory, University of California, Berkeley, CA 94720-3200 USA; 5 Present Address: Huazhong Agricultural University, Wuhan, China; 6 Present Address: University of California, Irvine, CA USA

**Keywords:** Cell death and immune response, Infection

## Abstract

Since the discovery of cell apoptosis, other gene-regulated cell deaths are gradually appreciated, including pyroptosis, ferroptosis, and necroptosis. Necroptosis is, so far, one of the best-characterized regulated necrosis. In response to diverse stimuli (death receptor or toll-like receptor stimulation, pathogenic infection, or other factors), necroptosis is initiated and precisely regulated by the receptor-interacting protein kinase 3 (RIPK3) with the involvement of its partners (RIPK1, TRIF, DAI, or others), ultimately leading to the activation of its downstream substrate, mixed lineage kinase domain-like (MLKL). Necroptosis plays a significant role in the host’s defense against pathogenic infections. Although much has been recognized regarding modulatory mechanisms of necroptosis during pathogenic infection, the exact role of necroptosis at different stages of infectious diseases is still being unveiled, e.g., how and when pathogens utilize or evade necroptosis to facilitate their invasion and how hosts manipulate necroptosis to counteract these detrimental effects brought by pathogenic infections and further eliminate the encroaching pathogens. In this review, we summarize and discuss the recent progress in the role of necroptosis during a series of viral, bacterial, and parasitic infections with zoonotic potentials, aiming to provide references and directions for the prevention and control of infectious diseases of both human and animals.

## Facts

- Regulated necrosis, including necroptosis, pyroptosis, and ferroptosis, can release damage-associated molecular patterns (DAMPs) which promote inflammation, a process termed necroinflammation.
- Necroptosis can be initiated by death receptor or toll-like receptor stimulation, pathogenic infection, or other stimuli.
- Pathogens like viruses, bacteria, and parasites evolved exquisite strategies to either inhibit or promote necroptosis for higher replication and persistent infection.

## Open questions

- What are the exact roles and detailed mechanisms of regulated necrosis during pathogenic infections of animals and humans? How these pathogens interact with the host immune system for better survival in hosts.
- Regulated necrosis is a double-edged sword, and can we precisely manipulate regulated necrosis as a potential therapeutic method in pathogenic infections, sepsis, Alzheimer’s disease, diverse stresses, and other relevant diseases and disorders?

## Introduction

Delicate control of cell proliferation and cell death plays a critical role during the development and homeostasis of multicellular organisms and during host defense against invading pathogens. There is no doubt that the breakdown of this delicate balance between cell proliferation and cell death can lead to physiological malfunction, organ damage, or even a variety of diseases in the body. In the past several decades, apoptosis has traditionally been considered the only form of programmed cell death, a gene-regulated active cell death. However, necrosis was considered an accidental form of cell death, an uncontrolled passive cell death [1]. However, increasing evidence shows that cell apoptosis is not the unique form of programmed cell death, and regulated necrosis (also termed necroptosis or caspase-independent cell death), pyroptosis, and ferroptosis have been successively identified by several groups [2–4]. Currently, necroptosis is one of the best-characterized regulated necrosis. Necroptosis is generally initiated through RIP3/RIP1 or RIP3/ZBP1 and subsequently executed by MLKL. Necroptosis requires the kinase activity of RIP3, a member of the family of the death-domain-containing kinase so-called “RIP1” [3, 5], and the downstream “executioner” protein MLKL [6]. RIP3 can be activated by one of the upstream proteins that contain the RIP homotypic interaction motif domain-like RIP1, TRIF (Toll/IL-1R domain-containing adapter-inducing IFN-β, also known as TICAM1), or DAI (also known as ZBP1) [7–10]. The role of necroptosis in a wild-type setting is not completely clear, but its activation pathways have been suggested as part of inflammation-mediated immunity during infection [11]. In this review, we summarize and discuss the recent research progress on the mechanism of necroptosis and its modulatory role in diverse zoonotic pathogen infections.

## Regulation of pyroptosis

**Fig. 1:**
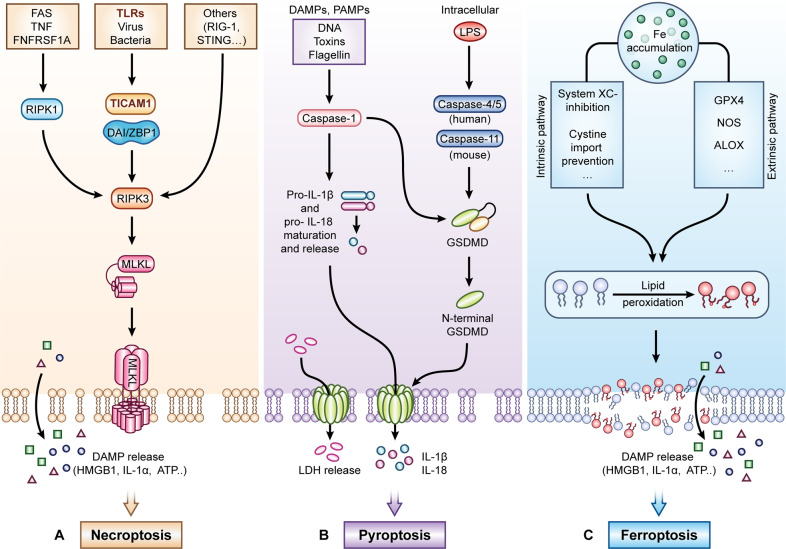
Molecular pathways of three kinds of regulated necrosis (Necroptosis, Pyroptosis, and Ferroptosis). **A** MLKL stimulation by RIPK3 is necessary for the execution of necroptosis. The initiators of necroptosis include death receptors (FAS and TNF), TLRs, viruses, bacteria, others (e.g., STING, RIG-1), and they activate RIPK1, TICAM1, DAI/ZBP1, or others downstream factors, leading to the RIPK3/MLKL activation, membrane rupture, and necroptosis. DAMP (e.g., HMGB1, IL-1α, ATP…) can be released during this process. **B** Cleavage of GSDMD mediated by active caspase-1 or caspase-11 (mouse) or caspase-4, 5 (human) is the executor for pyroptosis. Proinflammatory cytokines IL-1β and IL-18 were cleaved by active caspase-1. Caspase-1 or caspase-4, 5, 11 can be induced by upstream initiators, DAMPS, PAMPs, LPS, and others, leading to GSDMD cleavage, membrane rupture, and finally pyroptosis. **C** Ferroptosis, an iron-dependent regulated necrosis, is generally induced by lipid peroxidation. Ferroptosis can be triggered by extrinsic pathway (e.g., GPX4, NOS, ALOX) or intrinsic pathway (e.g., inhibition system XC-, prevention cystine import). The membrane is damaged, followed by DAMP release. TLRs Toll-like receptors, RIPK3 receptor-interacting protein kinase 3, MLKL mixed lineage kinase domain-like, DAI IFN-regulatory factors, also known as ZBP1, GSDMD gasdermin D, DAMPs damage-associated molecular patterns, PAMPs pathogen-associated molecular patterns.

Pyroptosis is a form of lytic-regulated cell death (RCD) first described in macrophages infected with the Gram-negative bacteria Shigella fexneri by Zychlinsky and colleagues in 1992 as apoptosis [12] but later renamed pyroptosis in 2001 by Cookson and Brennan [13] to reflect its inflammatory nature. Literately, pyroptosis is an inflammatory cell death caused by various stimuli like microbial infection and cancer, accompanied by activation of inflammasomes and maturation of proinflammatory cytokines interleukin-1β and interleukin-18 [14]. Pyroptosis plays a protective role in the host’s response to microbial infection [14–16] but also drives pathogenic inflammation [15, 16]. Distinct from apoptosis and necroptosis, pyroptosis is, in fact, the most immunogenic of all the cell death mechanisms. It has distinct morphological features such as cellular swelling, chromatin condensation, and plasma membrane permeabilization. Inflammasome activation was first coined in 2002 by Dr. Jurg Tshopp [14] and is a hallmark of pyroptosis. Inflammasomes are cytoplasmic multimeric protein complexes initiated by detecting PAMPs and DAMPs like DNA, bacterial flagellin, type 3 secretion system (T3SS) needle and rod subunits, and toxins which are detected by cytosolic sensors (NOD-like and RIG-I like receptor). The inflammasome formation leads to oligomerization of pro-Caspase-1 and pro-Caspase-11 (Caspase-4 and -5 in humans), resulting in the induced generation of their active form. Active Caspase-1 mediates the cleavage of gasdermin D (GSDMD), causing the release of the active form of N-terminal, which can induce cell death by pore formation through directly incorporating into the cellular membrane from inside [17] and outside of bacteria [18]. Proinflammatory cytokines IL-1β and IL-18 were cleaved by active caspase-1 during inflammasome activation. Distinct from caspase-1 mediated canonical inflammasome activation, Caspase-11 mediated noncanonical inflammasome activation is directly triggered by recognition of intracellular LPS and subsequently induces lytic cell death through cleaved gasdermin D [19, 20]. The executor of pyroptosis is mainly focused on gasdermin D, one of the Gasdermin family (GSDMs). Up to now, six genes have been classified as members of GSDMs based on their conserved N-terminal and C-terminal regions in humans, including gasdermin A (GSDMA), B (GSDMB), C (GSDMC), D (GSDMD), E (GSDME)/DFNA5, and DFNB59 [21]. All the members of the Gasdermin family, except DFNB59, are cleaved into two fragments and form pores on the cell membrane by the released N-terminal domain ensued by cell lysis [22, 23] (Fig. 1). In the context of pyroptosis, upon inflammasome activation, both GSDMD and GSDMB can be cleaved by caspase-1 [17, 24] and caspase-4 (in humans) [25, 26]. Of note, caspase-3, an important mediator of apoptosis, has been shown to cleave both GSDMB in human immune-related diseases [25, 27] and GSDME in antitumor studies, respectively [28–30]. While caspase-8, another important mediator of apoptosis, has been shown to cleave GSDMD [31] and GSDMB [31, 32] during Yersinia infection. These results further expand the understanding of the connection between pyroptosis and apoptosis through these functional proteins.

## Regulation of ferroptosis

Ferroptosis is a newly identified type of regulated cell death originally proposed by Dixon in 2012, and it is characterized by iron accumulation and lipid peroxidation. Ferroptosis is distinct from apoptosis, pyroptosis, and necroptosis in terms of morphological, biochemical, and genetic features [33–35]. Ferroptosis can be induced mainly by two pathways, either an intrinsic pathway or an extrinsic pathway. The intrinsic pathway is normally initiated by regulation of transporters (e.g., inhibiting system Xc-, preventing cystine import, activation of the iron transporters transferrin), and the extrinsic pathway is generally mediated by suppressing the intracellular antioxidant enzymes expression or activities (like GPX4, NOS, and ALOX) [36, 37] (Fig. 1). Some new regulators of ferroptosis are constantly reported, like FSP1-CoQ10-NADPH and BH4 [37]. The regulation of ferroptosis is quite a complex process. Numerous studies have shown that ferroptosis is closely associated with many diseases or disorders, including cancers, kidney, liver, gastrointestinal, and neurological disorders [33, 36, 37]. It is anticipated that manipulating the ferroptosis process, either activating or inhibiting, can be served as an effective therapeutic strategy to relieve the diseases described above. The regulatory mechanisms of ferroptosis are quite complex, and more gaps or hypotheses still need to be filled or confirmed.

## Mechanism of necroptosis

Unlike apoptosis featured by cell shrinkage, DNA fragmentation, and membrane blebbing, necroptosis exhibits cell swelling and rupture of the plasma membrane [38]. Necroptosis is a form of nonapoptotic cell death and can be initiated by a series of factors, including death ligands, microbial infection, Toll-like receptor (TLR), interferon (IFN), and sterile cell injury [2, 11, 39–42]. Given the importance of cell death during the host hemostasis, disease development, and host defense, our review will focus on the current progress illustrating the intricate role of necroptosis during host–pathogen (virus, bacteria, and parasites) interplay.

**Fig. 2:**
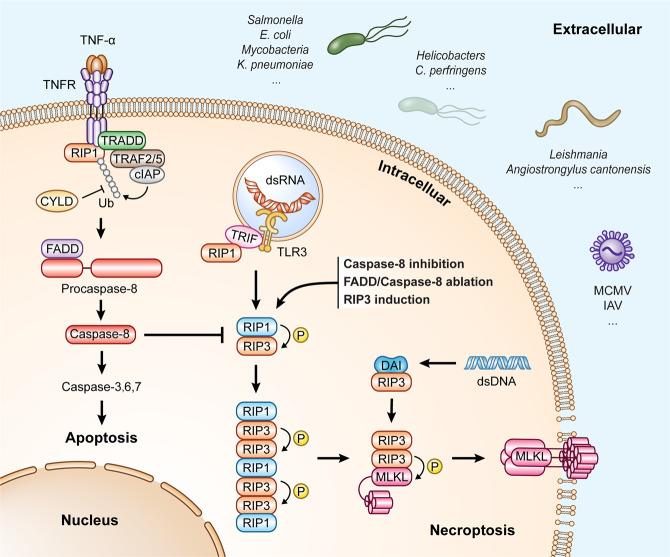
Regulation of cell apoptosis and necroptosis. Various stimuli, including the TLRs, IFN, death ligands, and pathogenic infections (Viruses, Bacteria, and Parasites), can induce cell death in necroptosis. TNF engages with its receptor and induces cell apoptosis by inhibiting cIAP or deubiquitination of RIP1 by cylindromatosis (CYLD). Instead, inhibition of FADD or caspase-8 can sensitize the cells to undergo necroptosis. Microbial infections can also initiate necroptosis via activation of the receptor-interacting protein kinase 1 (RIP1) and RIP3 complex. Further phosphorylation of MLKL by RIP3 can induce the oligomerization and translocation of MLKL to the membrane, eventually causing cell death. Besides the RIP1-RIP3-MLKL dependent-necroptosis, some RHIM-containing factors like TRIF and IFN-regulatory factors (DAI, also known as ZBP1) mediate the noncanonical necroptosis without the involvement of RIP1. TLRs Toll-like receptors, IFN interferon, FADD Fas-associated protein with death-domain, MLKL mixed lineage kinase do**ma**in-like, RHIM RIP homology interaction motifs (RHIM), ZBP1 Z-DNA-binding protein 1.

TNF is the best-studied extracellular death ligand among the diverse triggers for necroptosis. In 1988, Laster and colleagues showed that TNF induces target cells to undergo apoptosis and necrosis [43]. After TNF binding with its receptor TNFR1, multiple proteins are sequentially recruited to the cytosolic portion of TNFR1, thereby constituting a platform named “complex I” [44, 45]. Complex I can further activate the NF-kB and mitogen-activated protein kinases (MAPK), and the former is generally considered to initiate a survival signal [46]. Inhibition of cIAP or deubiquitination of RIP1 by cylindromatosis (CYLD) impedes the NF-κB pathway and hence induces cell apoptosis mediated by another platform, ‘complex II’ as named by some investigators [45, 47]. Genetic ablation or functional inhibition of caspase-8 or FADD can sensitize the cell to die in necroptosis instead of apoptosis, indicating that caspase-8 and FADD are crucial for guarding cells away from diverse cell death [48–50] (Fig. 2). In addition to necroptosis and apoptosis, caspase-8 also plays a role in inflammation-induced pyroptotic cell death, as shown by a recent study in mice expressing an enzymatically dead caspase-8 [51].

Cellular FLICE-like inhibitory protein (cFLIP), an antiapoptotic protein, is another important factor regulating the apoptosis pathway and necroptosis. cFLIP is mainly expressed as three isoforms in humans (cFLIP_L_, cFLIP_S_, and cFLIP_R_) and two isoforms in mice (cFLIP_L_ and cFLIP_R_) [52–54]. As described above, complex I normally activate the cellular survival pathway by activating NF-kB and cFLIP production. Generally, a sufficient cFLIP can prevent caspase-8 activation followed by inhibition of apoptosis, and insufficient cFLIP can fully activate caspase-8 leading to apoptosis activation. Insufficient cFLIP can prevent necroptosis via cleaving RIPK1 and RIPK3 [55]. Moreover, during the microbial infection, some viruses evolve a series of strategies to counteract the host protective machinery like apoptosis, and the data showed that several herpesviruses or poxviruses also express FLIP, sometimes termed vFLIP [56, 57]. vFLIP encoded by Kaposi’s sarcoma-associated herpesvirus can inhibit caspase-8 activation, thus preventing apoptosis and activating NF-kB [58, 59]. A recent report showed that mice constitutively expressing vFLIP showed excessive necroptosis in hepatocytes [60]. The data above indicates the critical functions of cFLIP in apoptosis and necroptosis, especially in the case of the TNF-mediated signaling pathway. Also, during the normal development of mice, cFLIP_L_ is extensively involved in inhibiting apoptosis and necroptosis from maintaining homeostasis. The precise roles of these isoforms of cFLIP during the regulation of cell survival, cell death, and normal mammalian development are still under investigation.

Upon the inhibition of apoptosis by pharmacological inhibitors or during microbial infection, necroptosis can be initiated through activation of the receptor-interacting protein kinase 1 (RIP1) and RIP3 complex, named “necrosome” (several researchers also call it “complex IIb”) [45, 61]. The main components of the necrosome complex are FADD, caspase-8, RIP1, RIP3, and TRADD. The presence of caspase-8 in the necrosome can prevent the interaction of RIP1 and RIP3 via the direct cleavage of these two kinases [50, 62, 63]. RIP1 and RIP3 interact through their respective C-terminal RIP homology interaction motifs (RHIM) [6]. Overexpression of RIP3 can also lead to necroptosis [64]. Several groups demonstrated that pseudokinase MLKL, a substrate of RIP3, is a downstream mediator for the execution of necroptosis. It is shown that phosphorylation of MLKL by RIP3 can further induce the oligomerization and translocation of MLKL to the inner leaflet of the membrane, subsequently causing a cellular rupture and final cell death (Fig. 2) [6, 65, 66]. Besides the RIP1-RIP3-MLKL dependent canonical necroptosis, some RHIM-containing factors like TRIF and IFN-regulatory factors (DAI, also known as ZBP1/DLM-1) mediate the noncanonical necroptosis without the involvement of RIP1 [10, 40, 67]. DAI can recognize the viral double-strand DNA or viral RNA, recruit RIPK3, and induce RIPK1-independent/RIPK3-dependent-necroptosis [9, 68, 69]. Tanshinol A, a natural compound, can induce reactive oxygen species production in lung cancer cells, which further activates necroptosis mediated by MLKL, independent of RIPK1 and RIPK3 [67]. Besides DAI, TRIF, another RHIM domain-containing protein, is also capable of activating RIPK3 independent of RIPK1 [10]. Under certain circumstances, as the inhibition of caspase, TLR3 or TLR4 stimulation induce necroptosis with the contribution of TRIF and RIP3 [10, 40, 70]. In addition to TRIF, studies from several groups demonstrated that RIP3, in combination with direct interaction with DAI plays a critical role in initiating RIP3-dependent-necroptosis during MCMV infection [71, 72]. Despite some initiators for necroptosis, most researchers agree that the eventual execution of necroptosis is universally driven by RIP3 and its substrate MLKL [11, 40, 65], although RIP3 was shown to induce apoptosis through its adapter function [73].

zVAD-fmk, a pharmacological inhibitor of caspase-8, has been extensively used to inhibit apoptosis from inducing necroptosis of cells [74]. Using a cell-based assay, Degterev and colleagues have successfully identified a small molecular necrostatin-1 (Nec-1) as an effective RIP1 inhibitor, and Nec-1 is capable of suppressing necroptosis [7, 75]. Nec-1 and genetic deficiency of RIP3 or MLKL have been widely applied to block the necroptosis pathway either in the cell model in vitro or in the knockout mouse model in vivo to decipher the mechanism of necroptosis.

## Immunological potentials of regulated necrosis-necroinflammation

Different from apoptosis without causing obvious inflammation, regulated necrosis, including necroptosis, pyroptosis, and ferroptosis, can release damage-associated molecular patterns (DAMPs), including HMGB1, IL-1alpha, DNA fragments, mitochondrial content, and ATP. Immune cells in an organism can respond to the exposed DAMPs, and this process can be generally defined as necroinflammation [76, 77]. In brief, DAMPs can further engage with the pattern recognition receptors (PRRs), like TLRs, and NODs, leading to the production of inflammatory cytokines via diverse inflammatory transcriptional pathways, including NF-κB signaling [78]. It is anticipated that certain diseases, especially those with overt inflammatory features (e.g., Crohn’s disease, multiple sclerosis, ischemia-reperfusion injury (IRI) in liver and kidney), may be relieved via inhibition of regulated necrosis. Research showed that inhibition of necroptosis via blocking RIPK1 kinase activity can protect against TNF-induced systemic inflammatory response syndrome in vivo [79, 80]. Notably, inhibiting regulated necrosis, including necroptosis and ferroptosis, showed an improved phenotype in a kidney IRI [81]. Regulated necrosis can be blocked and induced in some diseases, like cancers. It is well known that cancer cells exhibit resistance to cell apoptosis, necroptosis, and ferroptosis, showing promising performance in eliminating certain kinds of therapy-resistant cancer cells [80, 82, 83].

Moreover, in testing with various tumor types, RIPK3 activation can induce the expression of immunostimulatory cytokines, leading to an increased antitumor immunity [83]. Data showed that loss of GPX4 function either by genetic inactivation or pharmacological treatment could induce selective ferroptosis in cancer cells in vitro and also guard against tumor recrudesce in mice [84, 85], indicating that rationally manipulating ferroptosis or necroptosis or both may be an attractive way to treat certain kinds of cancers in clinic. However, we can not rule out the possibility that regulated necrosis can exhibit anti-inflammatory effects under some conditions. Although the exact links between regulated necrosis and clinical diseases are still unclear, some evidence from clinical settings and animal model research emphasizes the relevance of regulated necrosis in the diseases.

In response to certain stresses, like infection or tissue damage, hosts immediately take action to eliminate the foreign materials or restore tissue integrity via various defense mechanisms, including cell death, aiming to maintain body homeostasis [86, 87]. Dexamethasone, often utilized as an anti-inflammatory agent, is also one kind of stress. Several studies showed the potential of dexamethasone in modulating cell death, like regulated necrosis. Very recently, it has been proved that dexamethasone sensitizes erastin-induced ferroptosis via glutathione depletion [88]. Dexamethasone can also alleviate corneal alkali injuries by inhibiting the caspase-1/GSDMD-mediated pyroptotic signaling pathway [89]. Ballegeer *et al*. demonstrated that dexamethasone could inhibit TNF-induced-necroptosis in intestinal epithelial cells [90], indicating the potential application of dexamethasone in treating clinical diseases with the extensive involvement of ferroptosis and pyroptosis, like acute kidney injury, stroke, alkali injuries or others. In addition, Dexamethasone can induce cell apoptosis, necroptosis, and pyroptosis under certain pathophysiologic conditions in hosts [91–94]. The report showed that dexamethasone and SMAC mimetic cooperate to induce necroptosis dependent on RIPK3 and MLKL in lymphoblastic leukemia cells without caspase activation [94]. Dieckol can relieve dexamethasone-induced muscle atrophy via inhibiting NLRP3/GSDMD-mediated pyroptosis [92]. Thus, precisely manipulating regulated cell death can be served as a potential therapeutic method in diverse stresses.

## Necroptosis and microbial infection

During the past several decades, countless discoveries have been obtained through studying the host–pathogen interactions, like diverse evasion strategies utilized by crafty pathogens. Some discoveries have been applied to protect the host from infection. Traditionally, as non-inflammatory but programmed cell death, apoptosis is crucial in protecting the host from microbial infection without eliciting an excess inflammatory response. However, the recent progress of necroptosis has greatly drawn the researchers’ attention to study host–pathogen interplay.

It is known that dying or stressed cells can continuously release immunogenic endogenous molecular contents, DAMPs [78]. It is generally considered that apoptosis release no or very limited DAMPs. However, most researchers agree that necroptosis can release massive amounts of DAMPs, a strong inflammatory trigger to activate immune responses against pathogenic infections, as discussed above [77, 78].

A variety of pathogens, including viruses and bacteria, have evolved exquisite strategies to modulate cell apoptosis of host cell, including inhibiting apoptosis at the early stage of infection and promoting apoptosis at the late stage to subvert the host immune response for higher replication and persistent infection [95, 96]. What is the role of necroptosis during pathogenic infection? Is it beneficial or detrimental to the host? The following sections discuss the role of necroptosis during microbial infection and emphasize pathogenic infections.

## Viral infection

The host processes a variety of mechanisms to sense the invading virus to activate proinflammatory responses and induces cell death of infected cells for further clearance of viral pathogens. Thus programmed cell death, including apoptosis, necroptosis, and other types of cell death, is a vital component of host defense, although many viruses have evolved diverse strategies to subvert this host response.

## Murine cytomegalovirus (MCMV)

Both MCMV and human cytomegalovirus (HCMV) belong to herpesvirus, one kind of large and complex DNA virus. It is known that HCMV infection is very prevalent worldwide and can cause serious diseases, especially in the population with depressed or naïve immune responses [97]. Most members of cytomegalovirus have very strong host species specificity. HCMV can mainly infect humans or human cells, and MCMV often serves as an invaluable model for studying the pathogenesis of HCMV infection in humans [97, 98]. MCMV can trigger apoptosis, necroptosis, or both pathways to control the viral spread of even cross-species infection if certain cell death inhibitors are absent [39, 99–101]. In the meantime, MCMV has evolved many genes that exhibit immune-modulatory functions to manipulate the timing and ways of cell death. This sophisticated pathogen can express both the suppressors for apoptosis and inhibitors to prevent necroptosis [102, 103].

It was shown that MCMV could encode some viral inhibitors to prevent cell death, including cell apoptosis and necroptosis, by targeting diverse apoptotic and necroptotic pathways [72, 100, 104]. It is generally agreed that caspase-8 and Bak/Bax mediate the initiation and execution of cell apoptosis. One of the MCMV protein products, vICA, can bind to the pro-domain of caspase-8 and prevent its activation [105]; another viral protein, M36, can also target and block caspase-8 [100]. Similarly, HCMV also encodes several apoptosis inhibitors to modulate the host responses. HCMV UL36 (vICA, a homolog of M36) can restrain cell apoptosis via inhibiting caspase-8 activation [106]. Besides caspase-8 inhibition, HCMV pUL37x1 (vMIA) can also block the mitochondrial release of cytochrome C, resulting in the inhibition of caspase9-mediated cell apoptosis [107, 108]. Other viral proteins like vIBO and vMIA can function at the mitochondrial level and inhibit Bak and Bax, respectively [103]. Viral inhibitor of RIP activation (vIRA) encoded by the viral M45 gene can disrupt RHIM-dependent RIP3-RIP1 interaction, suppressing necroptosis [39]. It has recently been shown that mice deficient in Caspase-8 and RIPK3 mount elevated levels of CD8 T cells in response to MCMV infection, suggesting the cell death-independent role of Caspase-8 during restricting antiviral CD8 T cell hyperaccumulation [109].

Nevertheless, the role of RIPK3 and MLKL played during the restriction of antiviral CD8 T cell by Caspase-8 awaits further investigation. Besides the inhibitory effects of MCMV on the execution of necroptosis, MCMV infection can also indirectly trigger necroptosis of retinal neurons via inflammatory response mediated by ocular immune cells [110], clearly showing that the complex modulation of MCMV infection on host cell death. Lane et al. utilized an antiviral necroptosis-based CRISPR knockout screen to analyze this complex virus-host interaction and identified a critical host factor mediating early viral infection [111]. Similarly, HCMV can also block the necroptosis through the degradation of MLKL by UL36 [106, 112]. Therefore, HCMV UL36 is a dual cell death inhibitor for apoptosis and necroptosis. A recent study demonstrated that HCMV-induced autophagy could prevent cells from undergoing necroptosis [113], facilitating the viral spread in hosts. All of these studies with MCMV infection, either in cells or host body, unambiguously demonstrated that this virus could utilize diverse strategies to manipulate or subvert the immune response effectively, either leashing or delaying the proinflammatory reactions of cell death signaling to favor viral persistence in the host.

## Influenza virus

Influenza virus, an enveloped negative-sense and single-stranded RNA virus consists of types A, B, and C [114]. Influenza A virus (IAV) infection can cause severe diseases in mammals and birds through seasonal epidemics and occasional pandemics and pose a significant threat to human health, especially for populations with relatively compromised immune responses like young children and the elderly, and pregnant women [115].

Several studies showed that IAV infection could trigger programmed cell death, including apoptosis, necroptosis, or pyroptosis in various cell types, like macrophages, fibroblast, alveolar epithelial cells, and monocytes as in vivo animal models [69, 116–120]. Thapa et al. identified a host protein DAI which is originally considered to be a dsDNA sensor [71], and discovered that DAI could also sense genomic RNA of IAV and further induced RIPK3 independent apoptosis and parallel RIPK3-dependent-necroptosis in murine fibroblasts to eliminate the infected cells or tissue [69]. This group further found that compared to wild-type mice, RIPK3 knockout mice are more susceptible to IAV infection. This phenomenon is not observed in MLKL knockout mice. They demonstrated that apoptosis and inflammasome might also contribute to effectively controlling IAV infection besides necroptosis. A recent study showed that RNA from IAV can be recognized by ZBP1, leading to the activation of RIP3 and MLKL and following necroptosis [121–123], although the exact mechanism of how ZBP1 senses RNA is still under investigation. A study by Kuriakose showed that IAV infection in primary murine BMDMs could trigger multiple and parallel cell death pathways via this innate sensor, ZBP1/DAI [119]. The same group further identified a transcriptional regulator (IRF1) of ZBP1 promoting necroptosis during IAV infection [124].

Interestingly, Downey and colleagues identified a novel role of RIPK3 in regulating type I IFN antiviral immunity at both transcriptional and posttranscriptional levels during IAV infection, differing from its traditional role in necroptosis [125]. Recently, using a transgenic mouse model, Shubina et al. reported that necroptosis also plays a vital role in the antiviral host defense in the absence of apoptosis [126]. Conversely, RIPK3 deficiency can protect against Influenza H7N9 virus infection [127]. Thus, the exact role of necroptosis during the IAV infection is complex and dynamic, depending on infection stages, infection conditions, and others.

Few studies are available regarding the viral factors mediating necroptosis during viral infection. A study from Gaba et al. identified NS1 protein of IAV as a viral factor to interact with MLKL, leading to its oligomerization and eventually execution of necroptosis [128]. Besides the role of RIPK3 in regulating cell death and IFN secretion, RIPK3 also modulates the immune response of monocytes and DCs by promoting the expression of co-stimulatory molecules and T cell proliferation abilities [129]. This indirect modulation is mediated through the supernatant of necroptotic cells. These studies above provide concrete evidence that RIPK3 can exhibit distinct roles in different cell types, and the exact contribution of these pathways or viral factor(s) during IAV infection in vivo demands further investigation.

Besides IAV, animal-source influenza viruses emerged in the last decade and caused diseases in poultry, domestic animals, and human beings. These viruses include the best-known avian influenza virus (AIV) and swine-origin pandemic influenza virus [130, 131]. Taking AIV as an example, AIV mainly circulates in wild aquatic birds and domestic poultry but occasionally infects humans through direct and indirect contact with farmed poultry or migratory birds [132]. Both High pathogenic AIV H5N1 and low pathogenic AIV H7N9 can cause severe influenza diseases, even death in humans. Notably, H5N1 infection in humans generally causes a >50% fatality rate [133, 134], clearly manifesting their epidemic and pandemic potential and threat to public health. Little information is available regarding the role of necroptosis during AIV infection in animals and humans. Thus, understanding cell death, this key defense mechanism for the host during AIV infection in poultry or humans, await investigation and may promote early and quick diagnosis method and treatment.

## Sendai virus

Sendai virus (SeV) is an enveloped negative-strand RNA virus, and it mainly infects mice and rats, causing acute respiratory diseases resembling the human parainfluenza infection [135]. It was shown that SeV could induce cell apoptosis via activation of Caspase-8 and Caspase-3 [135]. The MVAS/MAPK kinase7/c-Jun N-terminal kinase 2 signaling pathway plays a role somehow [136]. SeV infection can also trigger dramatic induction of necroptosis in a RIG-1 (RNA sensor) dependent manner, and viral proteins Y1 and Y2 can sensitize the cells towards necroptotic death through degradation of cIAPs [137]. Interestingly, the viral protein Y2 has also been involved in antiapoptotic activities [138], showing that this versatile virus has adapted to counteract the host defense. Although SeV infection causes an augmented inflammation in RIP3 deficient mice, the exact role of necroptosis during different stages of Sev infection in vivo is not yet clear.

## Bacterial infection

### Salmonella

Gastroenteritis is the main cause of morbidity and mortality in humans, especially young children. *Salmonella* species are a leading cause of gastroenteritis [139]. Worldwide, typhoidal and nontyphoidal *Salmonella* infections have resulted in at least 118.3 million reported human cases with 355,000 deaths each year and posed a significant threat to human health and life [139–141].

*Salmonella* Typhimurium (*S*. Typhimurium), a typical Gram-negative intracellular enteric *Salmonella* species, is the etiologic agent of salmonellosis, and *S*. Typhimurium infection can cause gastroenteritis, septicemia, hepatitis, and even rapid death in humans [142]. Mouse inoculated with a very low dose (as low as 100 bacteria) of *S*. Typhimurium can die within a short period (as short as 7 days) [143]. More than 20 years ago, *Salmonella* infection could cause cell death, mainly apoptosis and accidental necrosis [144–146]. Recently, researchers realized that *S*. Typhimurium infection could induce necroptosis and pyroptosis in macrophages, the most crucial innate immune cells for *Salmonella* control [143, 147, 148]. Early efficient control of *S*. Typhimurium depends on the innate immune cells, mainly macrophages, and subsequent T cell response will be engaged late in the infection [142, 149]. Jorgensen et al. showed that *S*. Typhimurium infection in macrophages can induce the release of type I interferon, which further drives the necroptosis of macrophages in a RIP3/RIP1-dependent manner, contributing to evasion of host immune response [148], and another group discovered that intestinal epithelial Caspase-8 is essential to prevent *S*. Typhimurium infection induced-necroptosis [150]. Jorgensen’s group further demonstrated the link between type I interferon production and augmentation of necroptosis in *S*. Typhimurium infected macrophages are antioxidative stress response impairment via RIP3 [151]. At the same time, data from another group indicated that K45A medicates the kinase activities of RIP1 in the initiation of necroptosis in macrophages, and mice with K45A mutation in RIP1 exhibited attenuated inflammatory response and are highly susceptible to *S*. Typhimurium challenge [152]. It is well known that *Salmonella* pathogenicity island 2 (SPI-2)-encoded type III secretion system (T3SS), the most extensively studied and vital virulence factor of *Salmonella*, mediated the translocation of effector proteins into host cells, triggering proinflammatory responses or cell death [153, 154]. Several effector proteins, including SseK1, SseK3, and SpvB, inhibited cell necroptosis in infected macrophages and intestinal epithelial cells [155–157].

Moreover, TLRs, the widely studied PRRs to sensor the pathogen-associated molecular patterns (PAMPs), play a vital role in the pathogen recognition and induction of immune response during infections [158]. Ligands for TLR2, TLR4, TLR5, TLR9, and TLR11 are all present in *Salmonella*, which are lipoproteins, lipopolysaccharide (PLS), flagellin, CpG DNA, and flagellin, respectively [159–161]. Very limited reports illustrate the role of specific TLRs in regulating the necroptosis of host/cells during *Salmonella* infection, and Zhan et al. were the first to show TLR9 protects the host against necroptosis, preventing or delaying the systematic spend of the bacteria in the host [162]. Therefore, more research awaits further investigation, e.g., do other TLRs or NOD-like receptors play a role during necroptosis regulation in the cells/host and their contributions to disease development. Indeed, when caspases are inactivated in mouse macrophages, following recognition of poly(I:C) and LPS by TLR3 and TLR4, respectively, necroptosis can be induced with the mediation by TRIF [163]. Besides macrophages, neutrophils are another important cell type involved in bacterial control by the host [164]. Do neutrophils regulate necroptosis in collaboration with macrophages or independently? Intriguingly, premature cell death of neutrophils infected by *Brucella abortus* can promote phagocytosis by macrophages, favoring further bacterial replication in macrophages and in hosts [165], indicating the potential “cooperative work” between neutrophils and macrophages manipulated by evading pathogens.

Moreover, NleB, the effector protein of *Escherichia* (*E*.) *coli*, is capable of blocking cell death receptor signaling, including necroptosis, as discussed below [61], and NleB homologs are proved to present in *Salmonella* [166]. It needs to be determined whether *Salmonella* NleB is capable of modulating necroptosis. Further investigation of the *Salmonella* virulence factors during modulation of cell death would allow a better understanding of how hosts counteract bacterial infection. Recently, Doerflinger and colleagues showed that necroptosis coordinated with pyroptosis and apoptosis protects the hosts from *Salmonella* infection [167], indicating a complex defense mechanism hosts utilize in response to intracellular infections.

*Salmonella* causes persistent infection in humans and various animals, including poultry, pigs, cattle, and some pet animals (pigeons, fish, reptiles) [168–171]. Related production, carcass, or excretion from these animals with *Salmonella* infection or water/food contamination can serve as a potential bacterial reservoir for spread, and humans with direct or indirect contact with them could get infected. Cell death can cause bacterial spread into the environment leading to a public health concern. However, little information is available regarding the possible contribution of cell necroptosis to the pathology, bacterial shedding, and disease development during *Salmonella* infection in animals, especially farm and pet animals, and the responsible virulence factors during this process remain much to be uncovered.

### Mycobacterium

Tuberculosis (TB) is the ninth leading cause of death worldwide caused by *Mycobacterium tuberculosis* (*M.tb*). According to a WHO report, roughly 10.4 million people fell ill with TB globally, and 1.67 million deaths were estimated in 2016 (WHO, 2017). *M.tb* has co-evolved with human beings for tens of thousands of years, and several strategies have been evolved to efficiently manipulate host immune responses for better survival [172]. *M.tb* is an intracellular pathogen that generally infects and survives within the host macrophages and dendritic cells, neutrophils, and other non-professional phagocytes to a lesser extent [173–175]. Virulent *M.tb* can induce or inhibit cell apoptosis and can trigger necrosis of host macrophages to evade innate immunity and modulate the adaptive immune response [176, 177]. Currently, apoptosis and necrosis are the research focus for studying the host–pathogen interaction, and very limited studies are available about the modulation of necroptosis by this pathogen.

One report from 2013 shows that the TNF-RIP1-RIP3 axis mediates necroptosis of infected macrophages via mitochondrial reactive oxygen species (ROS) production [178]. Very recently, Butler and colleagues showed that *M.tb* infection and TNF-α synergize to induce necroptosis of murine fibroblasts via a RIP1-dependent manner in vitro and also the occurrence of necroptosis in granulomas in mice, possibly contributing to bacteria dissemination and transmission [179]. Another group confirmed that *M.tb* infection establishes a pro-necroptotic environment by up-regulating MLKL and ZBP1 and further demonstrated that macrophage necroptosis is eventually restricted to mitigate the disease outcome [180]. Several models of how *M.tb* induces necroptosis in macrophages have been established. Tuberculosis necrotizing toxin (TNT) can induce necroptosis in *M.tb*-infected macrophages through the release of ROS [181], and ROS could further promote necroptotic cell death in *M.tb*-infected macrophages. Although a subsequent study showed a contradictory conclusion, the discrepancy is possibly due to the difference in the cell line used or other unidentified reasons [182]. *M.tb* can also trigger macrophages necroptosis by consuming NAD by bacterial factor TNT [183]. Interestingly, the same group further discovered that the supply of NAD+ could reduce ROS levels in *Mtb*-infected macrophages and prevent cell death, contributing to the restriction of bacterial replication [184]. This study provides direction for better treatment of TB patients in the future.

It is unclear which other virulence factor(s) of *M.tb* modulate(s) the execution of necroptosis during the late stage of infection in vivo. The main genomic difference between the attenuated Bacillus Calmette-Guerin (BCG) and *M.tb* is the so-called region of difference 1 (RD1), which contributes to the full virulence of *M.tb* [185]. An intriguing report showed that the ESX-1 system and its substrate EAST-6, encoded by the RD1 region of *M.tb*, can induce necroptosis in macrophages dependent on inflammasome activation during bacterial infection [186, 187]. Thus it is worthy to further clarify the regulatory role of RD1 during the necroptosis of host cells during *M.tb* infection.

Besides human TB, zoonotic TB is another form of TB caused by *M. bovis* in humans. In 2016, an estimated 147,000 new human zoonotic TB cases and 125000 deaths were reported worldwide (WHO, 2017). Bovine TB can be transmitted from animals to humans through food consumption, usually raw or improperly cooked meat or dairy product from diseased animals and from humans to humans [188]. Currently, no report is available about the induction of necroptosis of host cells by *M. bovis* or its virulence factors. Quite early analysis using genomic subtraction showed that RD1 is present in all tested virulent *M.tb* strain and *M. bovine* strains [189], and it is reasonable to speculate some similarities between *M. bovine* infection and *M.tb* infection in the host, which needs to be further clarified.

### Helicobacter

*Helicobacter* (*H*.) *pylori* is a microaerophilic Gram-negative bacillus that infects more than half of the world’s population and is the best-studied Helicobacter species that can cause severe gastric diseases in human beings [190]. *H. pylori* has evolved diverse strategies and successfully adapted to human after colonizing the human stomach for at least 50,000 years [191]. Several virulence factors from *H. pylori* have been shown to cause cell apoptosis and necrosis, including Vacuolating cytotoxin A (VacA) and gamma-glutamyl transpeptidase (GGT) [192–194]. Persistent colonization of *H. pylori* in the host stomach leads to chronic inflammation, which further drives the occurrence of peptic ulceration and even gastric cancer. Radin reported that VacA might augment mucosal inflammation via inducing necroptosis of gastric epithelial cells and contribute to the pathogenesis of gastric diseases [195]. Due to the limited information, the exact role and contribution of necroptosis during *H. pylori* infection in vivo is still unclear, e.g., what is the disease outcome if RIP3 or MLKL is knockout in the mouse? Does necroptosis of gastric epithelial cells in the stomach play a protective or detrimental role during bacterial infection in the host? *H. pylori* infection in hosts is a complex and dynamic process. The effect of gastric epithelial cell necroptosis on disease outcome should be considered from different infection stages. Before the bacterial infection in the mucosa, necroptosis of gastric epithelial cells can trigger the immune response and expose the bacteria to the host immune environment. However, uncontrolled necroptosis may lead to excess inflammation and chronic infection as the disease continues.

Besides *H. pylori*, non-*H. pylori* helicobacters have also been detected in the human stomach; these bacteria can cause gastric diseases [196]. *H. suis* is the most prevalent gastric non-*H. pylori* helicobacters in humans and pigs are the natural host for this bacterium. Pigs and pork are most likely the main sources of human *H. suis* infection [196–198]. It was shown that *H. suis* could induce cell apoptosis and necrosis in the gastric epithelial cells and lymphocytes [194, 199]. However, no data is available about necroptosis’s involvement and possible contribution during *H. suis* infection in vitro and in vivo. More studies need to be done considering its zoonotic potential.

### Pathogenic *Escherichia* (*E.*) *coli*

Pathogenic *E. coli*, typically enteropathogenic *E. coli* (EPEC), uropathogenic *E. coli* (UPEC), Enteroaggregative *E. coli* (EATC), and Enterotoxigenic *E. coli* (ETTC), has been widely studied in humans, animals and food due to their ability to cause significant morbidity and mortality worldwide [200, 201]. It was reported that UPEC infection could induce RIP1/PIP3 dependent-necroptosis of macrophages via bacterial pore-forming toxin, and under the current experimental setup, necroptosis is proved to be the principal model of cell death. Intriguingly, they also demonstrated that other pore-forming toxin-producing bacteria exhibit similar effects on macrophages [202], highlighting the feasibility of counteracting these toxin-producing-bacterial infections and controlling disease outcomes via these pathways.

EPEC, another pathogenic *E. coli*, are important diarrheal pathogens for young children. Li and colleagues showed that EPEC NleB, a type III secretion system effector (T3SS), targets several death-domain-containing proteins in TNFR, FAS, and TRAIL death receptors complexes to block several host cell deaths, including necroptosis, making it the first identified bacterial virulence factor capable of hijacking the death receptor complexes to modulate host defense [61]. During EPEC infection, inhibition of proinflammatory reaction and apoptosis by diverseT3SS effector factors is one of the major mechanisms utilized by the bacteria to block antimicrobial host defense [203]. Recently, one group discovered that EspL, another cysteine protease effector of T3SS, can degrade the PHIM-containing proteins RIP1, RIP3, TRIF, and ZBP1/DAI to block necroptosis [204]. Although several T3SS effectors are shown to target the necroptosis machinery, they may function in different manners, clearly showing the complex features of host response modulation by pathogens. It is still unclear whether other bacteria, especially gastrointestinal bacteria, also possess NleB or EspL effector protein homologs and whether these effectors exhibit similar or different effects on host cell death signaling. Programmed cell death, including necroptosis and pathogens like EPEC, is always in an evolutionary race [205]. Thus exploring cell death mechanisms mediated by emerging or evolved bacterial virulence factors will be continually in progress. In addition, to our knowledge, no data is available about the involvement of necroptosis during EATC infection or ETTC infection.

### *Klebsiella pneumoniae*

*Klebsiella pneumoniae* (*K. pneumoniae*), a Gram-negative bacterium, is an important pathogen that can cause infection and severe disease in pulmonary and urinary tracts of humans globally, especially nosocomial pneumoniae and sepsis. Notably, *K. pneumonae* infection can be fatal in some patients with compromised immune conditions [206]. This bacterium has evolved several mechanisms to evade the attack of antibiotics or antimicrobial material. Due to its profound significance in clinical diseases, *K. pneumonae* has drawn the great attention of researchers and doctors.

*K. pneumonae* infection can cause host cells, especially macrophages and neutrophils, to die in diverse forms such as apoptosis, necroptosis, and pyroptosis [95, 207, 208]. To some extent, *K. pneumoniae* infection can inhibit or delay cell apoptosis or pyroptosis, leading to bacterial survival and further bacterial dissemination in the host, and this mechanism is utilized by some other pathogens as well. However, the mechanism or bacterial factor(s) that mediate cell death modulation is still unknown. In addition, one group showed that carbapenem-resistant *K. pneumoniae* could activate necroptosis in THP-1 cell lines, and further study using a transgenic mouse model indicates that necroptosis may contribute to the substantial immune dysregulation during *K. pneumoniae* infection [208]. Recently another group showed that *K. pneumoniae* could wisely subvert efferocytotic clearance of neutrophils by depressing cell apoptotic signatures and induction of necroptosis, demonstrating the ability of *K. pneumoniae* to modulate cell death to more inflammatory cell death [209]. The mechanism by which *K. pneumoniae* manipulates efferocytosis through cell apoptosis and necroptosis remains unidentified.

## Other bacteria

Other bacteria are also implicated in the induction and regulation of necroptosis in cell or animal models. *Clostridium perfringens* (*C. perfringens*) infection can cause histotoxic, enteric, or enterotoxemic diseases in animals and humans by producing many toxins [210]. β-toxin from *C. perfringens* type C strain can induce cell death of primary porcine endothelial cells. This observed cell death is inhibited by Nec-1, indicating regulated necrosis is most likely involved [211]. However, other experiments must be further performed to tamp this conclusion, e.g., involvement of RIP3, MLKL, and other necroptosis markers. A recent study demonstrated that a high level of enterotoxin from *C. perfringens* type C strain could induce necroptosis in Caco-2, Vero cells, and human enterocyte-like T84 cell lines [212]. These data showed that necroptosis is involved in cell death mediated by virulence factors, like diverse toxins, from *C. perfringens*. It is, however, necessary to elucidate the potential role, either beneficial or detrimental, of necroptosis during *C. perfringens* infection in vivo. Whether other toxins from *C. perfringens* also mediate the necroptosis needs to be explored, which can help determine if necroptosis induced in patients or animals can be targeted for clinical therapeutics.

## Parasitic infection

Some parasites can cause human diseases, like leishmaniasis, toxoplasmosis, malaria, and Chagas disease [213]. Leishmaniasis is often found in tropics and sub-tropics, and the etiologic agent is *Leishmania*, intracellular protozoa. A higher level of heme, an inducer for RIP1/RIP3/MLKL dependent-necroptosis, was observed in the leishmaniasis patients. However, RIP1 kinase regulates leishmania replication-independent heme-induced-necroptosis [214]. Angiostrongylus cantonensis infection can cause major nerve diseases, and both necroptosis and apoptosis occur in the brain tissue of mice infected by Angiostrongylus cantonensis [215]. *Toxoplasma gondii* (*T. gondii*), a typical, prevalent, and also the world’s most successful intracellular parasite, can asymptomatically persist in the central nervous system, causing devastating diseases in animals and humans. It was shown that CD8^+^ T cell-mediated immune response plays a vital role during *T. gondii* infection [216]. An early study showed that T cell-specific FADD^−/−^ mice succumb to T. gondii infection more easily than wild-type mice, underscoring the importance of keeping necroptosis in check-in T cells during parasite infection [49]. Interestingly, Ripk3^−/−^mouse exhibits a similar survival rate in response to *T. gondii*. However, Ripk3^−/−^ Casp8^−/−^ mice succumbed to *T. gondii* infection, indicating the vital role of Caspase-8 in controlling *T. gondii* infection in vivo [217]. Although studies with *T. gondii* show that necroptosis may not be crucial for the egress of *Toxoplasma gondii* [218], we still can not rule out the possible role of necroptosis during *Toxoplasma gondii* infection solely based on this cell model and experimental condition. Brief summary of necroptosis induced by common pathogens is described in Table 1.

**Table 1:** Brief summary of necroptosis induced by common pathogens described in this review.

Pathogen	Research models	Identified virulence factors involved	Effects on host	References
MCMV	Cell lines: NIH3T3 fibroblasts, 3T3-SA, SVEC4-10, MEFs, BMDMs; WT and transgenic mouse models	vICA, vMIA, M36, M45	Protective	[71, 100]
Influenza virus	Cell lines: BMDMs. Fibroblasts; WT and transgenic mouse models	NS1	Protective, but detrimental at severe conditions.	[69, 119, 128]
Sendai virus	L929	Y1, Y2	Proposed protective	[137]
*Salmonella*	Macrophages; WT and transgenic mouse models	K45A, SPI-2, NleB?	Detrimental	[151, 152, 157]
*Mycobacterium*	Fibroblasts, granulomas	ND	Detrimental	[179, 183, 184]
*Helicobacter*	AZ-521	VacA	ND	[195]
Pathogenic *E. coli*	293 T cells, Hela cells	NleB	Protective	[61]
*Klebsiella pneumoniae*	THP-1 cell line, WT, and transgenic mouse	ND	ND	[208]
*C. perfringens*	Caco-2, Vero cells, human enterocyte-like T84 cell lines	Enterotoxin, β-toxin	ND	[211, 212]
Leishmania	Bone marrow-derived macrophages	ND	Protective	[214]
*T. gondii*	WT and transgenic mouse	ND	Proposed protective	[49, 217]

## Role of cell death in sepsis

Dysregulated host responses to microbial infections can cause sepsis by hyper inflammation, life-threatening organ dysfunction, multiple organ failure, or even death if not treated properly [219]. World Health Organization’s first global report on sepsis showed that sepsis is the 3^rd^ cause of neonatal death, causing more than 11 million death each year. Diverse cell deaths, including apoptosis, necrosis, necroptosis, pyroptosis, and autophagy, can be induced during sepsis in infections. Comprehensive reports showed that these cell death pathways are frequently aberrantly-regulated, playing a vital role during the occurrence and progression of sepsis [220]. Altered apoptosis and necrosis are observed in sepsis and are closely associated with sepsis-induced organ dysfunction and increased inflammation [221, 222]. In certain cases, uncontrolled apoptosis sometimes can lead to a compromised immune response, which is related to a higher risk of secondary infection and the immunosuppressive pathophysiology of sepsis [223, 224]. Necroptosis is also involved in the sepsis progression. Inhibition of necroptosis via RIPK3 knockdown or Nec-1 treatment can relieve the inflammation and attenuate the symptoms of organ dysfunction in septic mouse, zebrafish, and piglet models [225–228]. Evidence demonstrated that excessive pyroptosis also contributes to sepsis progression. Inhibition or disruption of pyroptosis can protect hosts against sepsis [229, 230].

**Fig. 3:**
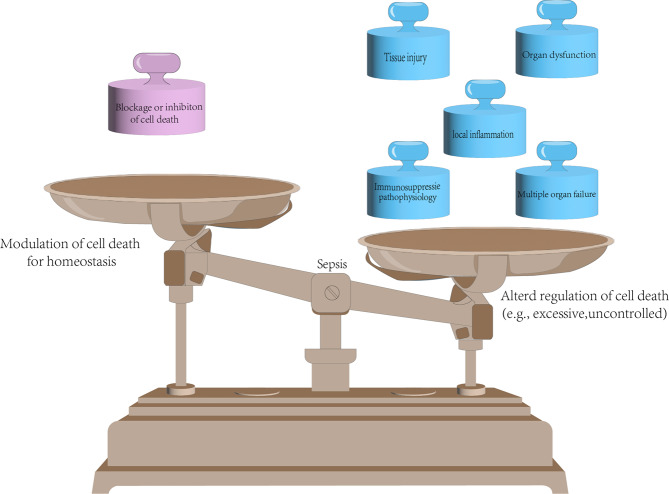
Altered regulation of cell death in sepsis. Severe infections can cause sepsis, and apoptosis, necrosis, necroptosis, pyroptosis, and autophagy, can be induced during sepsis. Altered regulation of cell death can lead to local inflammation, tissue injury, organ dysfunction, immunosuppressive pathophysiology, and multiple organ failure. Uncontrolled cell death can be manipulated to restore homeostasis.

Interestingly, Chen et al. discovered that the synergetic effects of RIPK3-mediated necroptosis and GSDMD-mediated pyroptosis contribute to the multiple organ injury of sepsis in mice, and blockage of both pathways can protect mice from lethal sepsis [33, 231]. Ferroptosis is another form of cell death discovered in sepsis, and evidence shows that treatment with a ferroptotic inhibitor can mitigate the LPS-induced sepsis [227, 232], indicating that targeting ferroptotic cell death is another way to counteract the sepsis (Fig. 3). Overall, regulated necrosis is a double-edged sword, it can protect hosts from pathogenic infections, but on the other hand, excessive execution is also inevitably capable of leading to sepsis. Therefore, modulating these cell death signaling pathways in the future may provide new therapeutic directions for sepsis treatment.

## Concluding remarks

Necrosis is not accidental anymore; it can be highly regulated and genetically controlled. Although regulated necrosis has been studied for more than 20 years, the term necroptosis was coined by the group of Prof. Junying Yuan in 2005. The pathways of necroptosis are well characterized, and the regulatory role of necroptosis in microbial infection has also been partially revealed. Necroptosis is implicated not only during microbial infection but also in various diseases, such as Alzheimer’s disease, neurodegenerative disorders, acute kidney injury, atherosclerosis, severe inflammatory response syndrome, and cancer. The precise mechanism and pathophysiological significance of necroptosis in diseases of humans and animals still need further in-depth investigation.

## Data Availability

All data in this study are included in this published article, and additional information are available from the corresponding authors upon reasonable request.
